# Characterization and Antioxidant Activity of Mannans from *Saccharomyces cerevisiae* with Different Molecular Weight

**DOI:** 10.3390/molecules27144439

**Published:** 2022-07-11

**Authors:** Yingyuan Zhao, Jiaqi Wang, Qianzhen Fu, Huiru Zhang, Jin Liang, Wenjie Xue, Guoqun Zhao, Hiroaki Oda

**Affiliations:** 1 College of Biological Engineering, Henan University of Technology, Zhengzhou 450001, China; yyzhao2009@outlook.com (Y.Z.); 13213145119@163.com (J.W.); fu13663843122@163.com (Q.F.); lj18535002481@163.com (J.L.); 2 Laboratory of Nutritional Biochemistry, Graduate School of Bioagricultural Sciences, Nagoya University, Nagoya 464-8601, Japan; hirooda@agr.nagoya-u.ac.jp; 3 School of International Education, Henan University of Technology, Zhengzhou 450001, China; baiqi158555@outlook.com; 4 College of Food Science and Biology, Hebei University of Science and Technology, Shijiazhuang 050018, China; gqzhao18@126.com

**Keywords:** mannan, molecular weight, antioxidant activity

## Abstract

Polysaccharides were extracted from natural sources with various biological activities, which are strongly influenced by their chemical structure and molecular weight. In this research, mannans polysaccharides were obtained from *Saccharomyces cerevisiae* by ethanol precipitation. The molecular weight of YM50, YM70, and YM90 mannans was 172.90 kDa, 87.09 kDa, and 54.05 kDa, respectively. Scanning electron microscopy of YM 90 mannans showed a rough surface with numerous cavities, while the surfaces of YM50 and YM70 were relatively smooth. Sepharose CL-6B and FTIR indicated that mannans had the characteristic bands of polysaccharides. The antioxidant activities of polysaccharides were evaluated in vitro using various assays. Mannans showed a good scavenging activity of DPPH radicals which depend on the molecular weight and concentration, and a higher scavenging activity of hydroxyl radical than ferric-reducing power activities. For the three types of mannans, cytotoxicity and hemolytic activity were rarely detected in mice erythrocytes and Caco-2 cells. Those results could contribute to the further application of mannans from *Saccharomyces cerevisiae* in the food and medicine industry.

## 1. Introduction

Polysaccharides widely exist in microorganism, plants, and animals. They are very important biomacromolecules in life activities and play critical roles in cell–cell communication and molecular recognition in the immune system. *Saccharomyces cerevisiae* (Brewer’s yeast) is described as a generally recognized as safe (GRAS) microorganism, and also contains nutritional value and bioactive properties [1,2]. Mannans are essential structural components of the yeast cell wall, and are generally associated with proteins in the outer and inner membrane. Mannans were isolated by extracting whole yeast cells and isolated cell walls, followed by precipitation with Fehling’s solution and a high temperature and pressure. Cell wall enzymatic hydrolysis with proteases and glucanases were used to extract mannan. Furthermore, some conventional technologies have also shown potential applications, such as solvent precipitation, ultrasound, and pulsed electric field. Characteristically, *S. cerevisiae’s* mannans are composed of long D-mannose chains linked by *α*-(1–6) bonds backbone, with short side-chains in the *α*-(1–2) and *α*-(1–3) linkages [3]. Mannans are also present as small oligomannosidic units (mannan oligosaccharides (MOS)) with *α*-(1–2) and *α*-(1–3) linkages that are linked to serine or threonine residues. Mannans form yeast, and present several commercial applications, relevant health benefits, and specific bioactivities, which tailor their potential use in pharmaceutical industries [4,5].

Yeast mannans exhibit various health benefits, such as immunomodulatory activity, radical-scavenging abilities collateral sensitivity, prebiotic activity, etc. [1]. These nutritional and bioactive properties have increasingly attracted the attention of the food, pharmaceutical, and cosmetic industries. This has led to many developments in their molecular structure, biocompatibility evaluation, and bioactive mechanisms. Moreover, polysaccharides are usually composed of various monosaccharides, and some have hyperbranched structures. Polysaccharides that have high molecular weights tend to form aggregates in solution. Although these crude polysaccharides have diverse biological functions, their high molecular weight (MW) leads to their low solubility and limited application. It has been reported that multiple polysaccharide bioactivities could be significantly associated with their molecular weight [6]. Some studies have found that polysaccharides with a lower MW have better antioxidant activity [7,8]. However, further data from the literature are available on their pharmacological and biological activities, as well as the relationships between these activities and the molecular weight of mannans from the *Saccharomyces cerevisiae,* have not been reported.

In this research, three mannan polysaccharides with different MWs were isolated and purified from the *Saccharomyces cerevisiae* yeast, and their molecular characterizations were preliminarily characterized by scanning electron microscope (SEM), size exclusion chromatography (SEC), and Fourier transform infrared spectroscopy (FTIR). Then, the mannans’ antioxidant activity was evaluated by various methods in vitro. Subsequently, cytotoxicity and hemolysis were evaluated by Caco-2 cells and mice red blood cells, respectively. Our study provided new perspectives for the possible molecular mechanisms and the utilization of mannans from *Saccharomyces cerevisiae* in food and medicine fields.

## 2. Materials and Methods

### 2.1. Materials

Mannans extracted from *Saccharomyces cerevisiae* with different molecular weights (YM50, YM70, and YM90) were supplied by Guoqun Zhao’s group (Hebei University of Science and Technology, Shijiazhuang, China). Caco-2 cells (ATCC, HTB037) were obtained from American Type Culture Collection. Dulbecco’s modified Eagle’s medium (DMEM) was purchased from Gibco Laboratories (Lenexa, KS, USA). Dextran standard, dextran blue 2000, nonessential amino acids (NEAA), fetal bovine serum (FBS), trypsin-EDTA, and 3-[4,5-dimethylthiazol-2-yl]-2,5-diphenyl tetrazolium bromide (MTT) were purchased for Beijing Solarbio Chemical Co. (Beijing, China). Other chemical agents were analytical grade and used directly, without further treatment, unless otherwise specified.

### 2.2. Scanning Electron Microscope (SEM)

The morphological structures of mannan samples were observed with a Quanta 250 FEG Scanning Electron Microscope (Thermo scientific, Waltham, MA, USA) as previously reported [9]. Based on the results of preliminary experiments, mannan samples with different molecular weights were prepared by being immediately pulverized into powder. The electron microscopy was set at a 5 kV accelerating voltage.

### 2.3. Molecular Weight Characterization of Mannans

The molecular weight (MW) of mannans (YM50, YM70, and YM90) was investigated. The protein-free polysaccharides were dissolved in distilled water. Then, the supernatant was sequentially applied to Sepharose CL-6B eluted at a flow rate of 0.8 mL/min with distilled water, 0.05, 0.1 and 0.2 mol/L NaCl solutions. A total of 2 mL of blue dextran 2000, a polysaccharide that is covalently bonded with blue dye, was injected and run with a flow rate of 0.8 mL/min. The automatic fraction collector was applied to collect a fixed volume of 4.5 mL fractions. Each fractionated eluate was collected by a fraction collector, and the carbohydrate content of each tube was monitored at 490 nm by phenol–sulfuric acid assay. Different-molecular-weight Dextrans (T3, T10, T70, T100, and T300) were used to establish a standard curve, following the method described by Luo et al. [10]. The K_av_ values for mannans were calculated according to Ackers’ formula [11]

K_av_ = (V_e_ − V_0_)/(V_t_ − V_0_) (1)

where V_0_ is void volume, V_e_ is total bed volume, and V_t_ is elution volume. In this method, these samples were determined by 2 mg/mL blue dextran 2000 (MW 2000 kDa) and cytochrome C, respectively. The absorbances for blue dextran 2000 and cytochrome C were monitored at 206 nm and 412 nm, respectively. The elution volume (V_e_) was calculated by estimating the principal peak in polysaccharides. The molecular weight of mannans before and after acidification was estimated using the standard curve.

### 2.4. FTIR

Infrared spectra of mannans samples were recorded with a Fourier transform infrared (FT-IR) spectrometer Nicolet iS50 (Thermo scientific, Waltham, MA, USA). The scanning wave was within 400–4000 cm^−1^ to detect functional groups, and all spectra were baseline-corrected according to a previous method [12].

### 2.5. Measurements of Free Radical Scavenging Activity of Mannans

#### 2.5.1. Measurements of Reducing Power of Mannans

The reducing power assay was determined by testing the reducing power of iron using the method by Slima et al. [13]. The reaction involves 1.0-mL mannan samples at different concentrations, 2.5 mL phosphate buffer (0.2 M, pH 6.6) and 2.5 mL of 1% (*w*/*v*) potassium ferricyanide. After incubation at 50 °C for 20 min, the reaction was stopped by adding 2.5 mL of trichloroacetic acid (10%). The supernatant (3.0 mL) was mixed with 3.0 mL of distilled water and 0.5 mL of ferric trichloride (0.1%) and then incubated for 10 min. The absorbance was measured at 700 nm.

#### 2.5.2. Measure of DPPH Radicals Scavenging Activity of Mannans

The antioxidation properties of mannans with different molecular weights were assessed by radical scavenging effects on 1,1-diphenyl-2-picrylhydrazyl (DPPH) free radicals, according to the previously described protocol [14]. First, DPPH was dissolved in a methanol solution to obtain a 100-μM solution for further use. Then, 2.0 mL of the mannans sample solution was added to 2.0 mL of DPPH solution and ascorbic acid was used as the positive control. Next, the mixtures were shaken and incubated in the dark at 25 °C for 20 min. Subsequently, the absorbance of these mixtures was measured at 510 nm using a UV-Vis spectrophotometer at different time intervals. Finally, the free radical scavenging efficiency was calculated according to the following equation:

DPPH scavenging activity (%) = [A_0_ − A_i_ + A_j_]/A_0_ × 100% (2)

in which A_0_ stands for the absorption of the blank solution (water + DPPH); A_i_ and A_j_ indicate the absorption of the sample solution (methanol + mannans + DPPH) and solvent solution (absolute ethyl alcohol), respectively.

#### 2.5.3. Measure of Hydroxyl Radicals Scavenging Activity of Mannans

Mannans’ capacity to scavenge hydroxyl radicals was measured according to Luo, et al. [10], with slight modifications. In brief, the reaction mixtures contained phosphate buffer (2.0 mL, 6.0 mM, pH 7.4), 1,10-phenanthroline (2.0 mL, 6.0 mM), ferrous sulfate (2.0 mL, 6.0 mM), and mannans sample solution (2.0 mL, 0.05–1.6 mg/mL) in a 10 mL colorimetric tubes. Hydrogen peroxide (1.0 mL, 0.01%, *v*/*v*) was then added and the mixtures were incubated at 37 °C for 60 min. The hydroxyl radicals were measured by monitoring the absorbance at 510 nm. Deionized water was used as the blank. The capacity to scavenge hydroxyl radical was calculated using the following formula:

Hydroxyl scavenging activity (%) = [A_0_ − A_i_ + A_j_]/A_0_ × 100% (3)

where A_0_ is the absorbance of blank, A_i_ is the absorbance of mannans samples, and A_j_ is the absorbance of the control solution (de-ionized water instead of sample and H_2_O_2_).

### 2.6. Cytotoxicity of Mannans in Caco-2 Cells

To investigate the biocompatibility of mannans with various molecular weights, the 3-(4,5-dimethylthiazol-2-yl)-2,5-diphenyltetrazolium bromide (MTT) assay was used to evaluate their cytotoxicities against Caco-2 cells, as the previous method [15]. In brief, Caco-2 cells in their logarithmic growth phase were seeded in 96-well plates at a seeding density of 8000 cells/well in 100 μL medium. After the cells adhered to the plates, the culture medium in each well was replaced with 200 μL of the test mannan samples (mannan solutions with a final concentration ranging from 10 to 160 μg/mL in complete DMEM medium). Cells were incubated for 24 h and then the viability of the cells was determined using MTT assay. The absorbance of each well was measured at 490 nm with a microplate reader (Bio-Rad, Hercules, CA, USA).

### 2.7. Determination of Hemolytic Activity

Hemolytic activity of mannans on mice eyeball erythrocytes was measured by a modified method according to Slowing [16]. In brief, freshly collected mice (SPF grade, KM) eyeball blood samples were mixed with an anticoagulant solution of heparin. To obtain a pure suspension of erythrocytes, the blood sample was washed three times in five volumes of sterile NaCl (0.9%) saline solution. After each washing, cells were pelleted by centrifugation (3000 rpm for 10 min at 4 °C). The supernatant was then removed by gentle aspiration. Erythrocytes were finally resuspended in phosphate buffer saline (PBS) to create 1% solution for hemolytic assay. Different-molecular-weight mannans (YM50, YM70, and YM90) were added to the suspension of red blood cells to obtain the final concentrations of mannans (80–200 μg/mL). The mixtures were incubated at 37 °C for 30 min in a water bath and then centrifuged at 3000 rpm for 5 min at 4 °C. Positive control and negative control were also run by incubating erythrocytes with distilled water and 0.9% NaCl saline solution, respectively. The absorbance of the supernatant was measured at 540 nm to measure the extent of red blood cell lysis using the following equation:

Hemolysis (%) = (A_0_ − A_balnk_)/(A_control_ − A_balnk_) × 100% (4)

where A_0_, A_balnk_ and A_control_ were the sample absorbance, the absorbance of negative control, and the absorbance of positive control, respectively.

The animal study was approved by the Animal Care Committee of Henan University of Technology (SCXK 2017-0001), China. All surgical procedures were performed under isoflurane anesthetization, and all efforts were made to minimize suffering.

### 2.8. Statistical Analysis

Statistical data analysis was performed using Microsoft Excel 2013 software (Redmond, WA, USA) and IBM SPSS Statistics software 22.0 version. Statistical comparisons were performed by using one-way analysis of variance (ANOVA) and Tukey’s test, and *p* < 0.05 was considered statistically significant.

## 3. Results and Discussions

### 3.1. Morphology of Mannans

**Figure 1:**
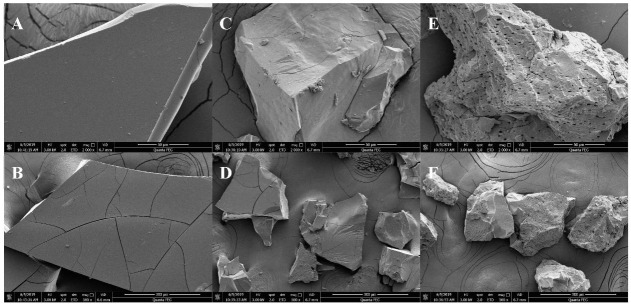
Scanning electron micrographs of mannans with different molecular weights ((**A**–**F**) stand for YM50, YM70, and YM90, respectively).

SEM technology was used to compare the spatial structure of mannans with different molecular weights (MW), namely, YM50, YM70, and YM90. To better realize the impact of spatial structures on the physical and chemical properties of mannans, the structure and surface topography of polysaccharides could be influenced by different purification and molecular weights. The microstructure of mannans was determined by SEM analysis and the obtained surface morphology is shown in Figure 1A–F. Cross-sectional SEM results showed that the 3D images of mannans were gobbet-like or had a honeycomb morphology with irregular shapes and sizes. Figure 1A,B showed that the high-MW YM50 mannans appeared as a relatively smooth sheet. As shown in Figure 1C,D the middle-MW YM70 mannans were rubbly with crisscrossing cracks. However, YM90 mannans in Figure 1E,F had a rough surface with numerous cavities. The pore size of mannans significantly increased as the MW increased. This was suggested to be related to the intermolecular and intramolecular forces. These findings are similar to previous works studying polysaccharides [17,18]. These differences in microstructure allowed for greater exposure and facilitated their utilization in different application fields.

### 3.2. Molecular Weight of Native Mannans

**Figure 2:**
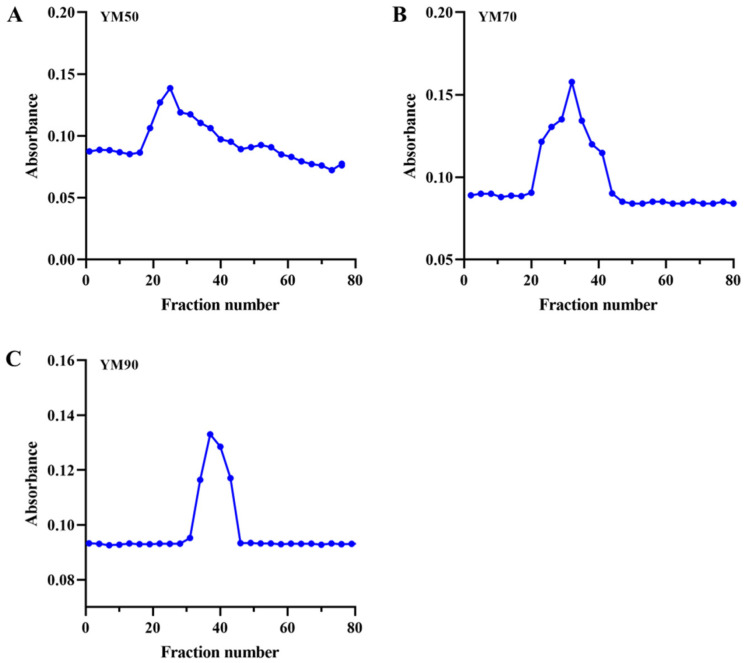
Sephadex CL-6B gel filtration column chromatogram of mannans. (**A**–**C**) were the elution profile of YM50, YM70 and YM90 mannans, respectively.

The crude polysaccharides extracted from *Saccharomyces cerevisiae* was dissolved in distilled water and a preliminary separation was conducted. To verify the molecular weight of polysaccharides, the mannans were further separated and sequentially purified through a cross-linked Sephadex CL-6B gel filtration column. Molecular size-based size exclusion chromatography (SEC) is a widely used technique for the determination of the molecular size distribution of polymeric materials (proteins, polysaccharides, and nucleic acids) [19,20]. The chromatogram indicated that the peak in YM50, YM70, and YM90 polysaccharides started from 15, 20, and 25 fraction tubes, respectively (Figure 2, blue-dotted). The solution profile of the YM70 and YM90 mannans showed an asymmetrical single peak, indicating that the polysaccharide was a homogeneous component (Figure 2B,C blue-dotted), while the profile of YM50 mannans showed a relatively wide molecular weight distribution (Figure 2A, blue-dotted). The K_av_ values for native YM50, YM70, and YM90 were calculated using Acker’s formula [21] and the values were found to be 0.0917, 0.209, and 0.294, respectively (Figure 2 and Table 1). Table 1 shows the relative percentages of the three mannans and elution fractions in different-molecular-weight divisions. The molecular weights (MWs) of YM50, YM70, and YM90 mannans were calculated to be 172,902 Da, 87,096 Da, and 54,050 Da, respectively (Table 1). In short, obvious differences could be seen in the molecular weight distribution of mannans polysaccharides.

### 3.3. Molecular Structure of Mannans

**Figure 3:**
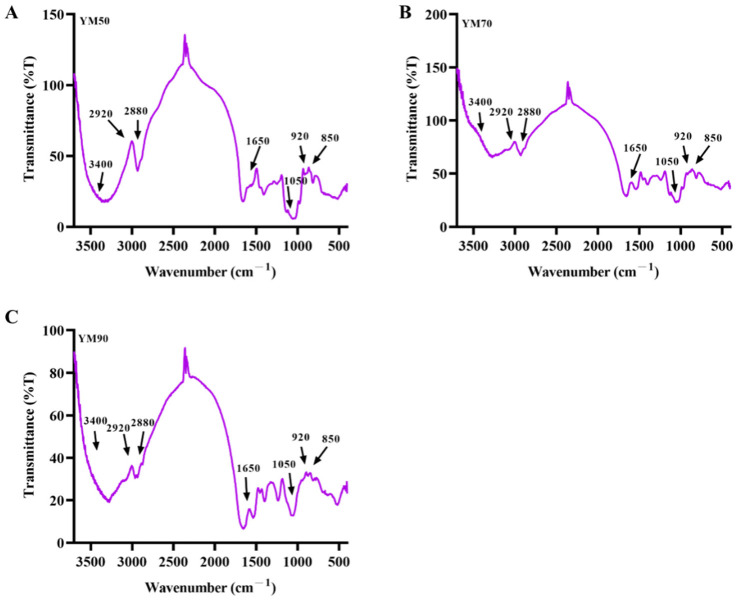
FTIR spectra of mannans ((**A**–**C**) stands for YM50, YM70, and YM90, respectively).

The FTIR spectrum of mannans is depicted in Figure 3, and no notable differences can be observed in the functional groups of mannan with various MW. The FTIR spectra of the mannans samples were similar, with slight differences in intensity. The absorption peaks in mannans YM50, YM70, and YM90 at around 3400 cm^−1^ belonged to -OH stretching vibrations in the glycosidic structure. The bands in the region of 2920 cm^−1^ and 2880 cm^−1^ were due to the methyl and methylene -CH stretching and bending vibrations, indicating that mannans have the characteristic peak in polysaccharides. The absorption peaks from 950 to 1200 cm^−1^ were generally recognized as the “fingerprint” region, which belonged to the stretching vibration of typical bonds in carbohydrates, such as C-CO and CO-H [22]. The absorption bands in the region of 1050 cm^−1^ suggest a pyranose ring of saccharides. Absorption at 920 and 850 cm^−1^ are typical for the α-dominating configuration in the pyranose of mannans [23,24]. Based on these results, mannans from *Saccharomyces cerevisiae* exist as a structure consisting of the α-dominating configuration in pyranose sugar, which is in accordance with the previous study [24].

### 3.4. Antioxidant of Mannans In Vitro

Many methods are recommended to provide a comprehensive assessment of the antioxidant property of polysaccharides. Here, the antioxidant efficacy of mannans with different MWs was evaluated using various antioxidative assays.

#### 3.4.1. Effect on Ferric Reducing Power

**Figure 4:**
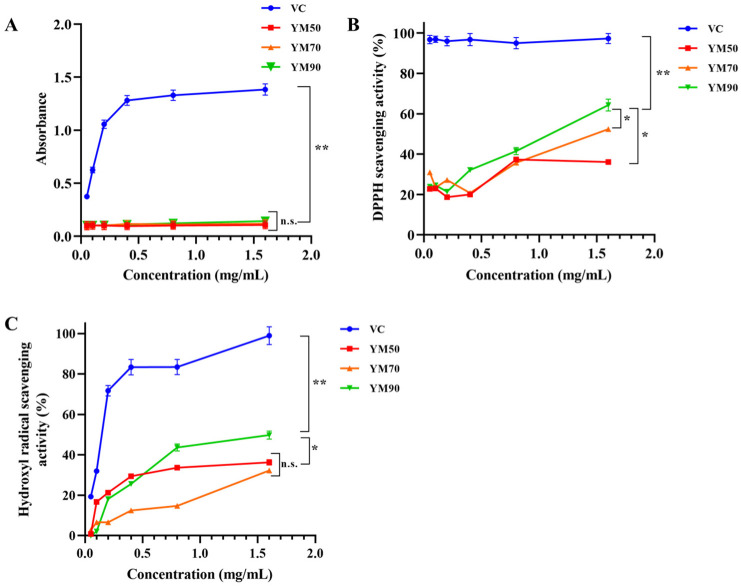
Scavenging effect on reducing power (**A**), DPPH free radical (**B**), and hydroxyl radical (**C**) of mannans at different concentrations. Vitamin C was used as positive control. All analyses were carried out in triplicate, and * *p* < 0.05, and ** *p* < 0.01, n.s.: no significant difference.

The reducing capacity of natural polysaccharides may serve as a significant indicator of their potential antioxidant activities. In this study, we investigated Fe^3+^-Fe^2+^ reductions, considering that measuring the formation of Perl’s Prussian blue at 700 nm can help to monitor the Fe^2+^ concentration. The absorbance of the sample solution at the wavelength of 700 nm was high when the antioxidant ability was strong. The reducing powers of mannans, as well as Vitamin C, are shown as a function of their concentrations in Figure 4A. The reducing power of Vitamin C linearly increased and there were significant differences between Vitamin C and mannans (*p* < 0.01). Vitamin C’s reducing power increased with the increase in concentration and reached a plateau of 1.33–1.38 (OD 700 nm) at a dose of 0.80–1.60 mg/mL, respectively. However, mannans with different MWs (YM50, YM70, and YM90) showed a similar lower reducing ability compared to that of the control sample at all tested concentrations.

#### 3.4.2. Effect of Scavenging on DPPH Radicals

DPPH is a free radical compound that has been widely used to determine the free radical scavenging abilities of various substances. The total DPPH scavenging effects of all mannan samples and vitamin C at varying concentrations were detected by measuring the decrease in DPPH radical absorbance at 510 nm (Figure 4B). In this report, a medium DPPH scavenging activity was observed for mannans at 0.10 mg/mL, with a percentage elimination of 23.80, whereas vitamin C showed a minimum percentage elimination of 96.80. No statistically significant difference was observed in the three types of mannans at relatively low concentrations (0.05–0.20 mg/mL). In the concentration range from 0.20 to 1.6 mg/mL, the scavenging capacity on DPPH was dramatically increased with increasing concentration. The DPPH radical scavenging activities of YM50, YM70, and YM90 mannans at maximum concentration 1.6 mg/mL were 36.10%, 52.40%, and 64.30%, respectively, and there were significant differences between them (*p* < 0.05). Polysaccharides with a lower MW may have better water solubility, and could more easily react with many free radicals. The free-radical quenching potential of polysaccharide mannans could be due to the presence of the carboxyl group [10]. Our findings are in accordance with previous research results presented by Zheng et al. [25], who reported that polysaccharides’ antioxidant activities were related to their molecular weight, glycosidic linkage, and sulfation degree.

#### 3.4.3. Effect of Scavenging on Hydroxyl Radicals

Hydroxyl radicals can easily cross cell membranes, and readily react with carbohydrates, proteins, lipids, and DNA, before causing tissue damage or cell death [26]. Hence, scavenging hydroxyl radical is important for antioxidant defense. The scavenging ability of mannans at different hydrogen peroxide concentrations is shown in Figure 4C and compared with that of vitamin C. The scavenging ability of all tested samples (including YM50, YM70, YM90 mannans, and vitamin C) for hydroxyl radicals significantly increases as the concentration increases, at 0.05–01.6 mg/mL. The antioxidant capacity of mannans and vitamin C shows a dose-dependent scavenging activity. The scavenging effects of YM50, YM70, YM90 mannans, and vitamin C on hydroxyl radicals at a concentration of 1.6 mg/mL are 36.30%, 32.30%, 49.80%, and 99.01%, respectively. YM90 mannans showed high antioxidant activity, even though their scavenging activities were lower than that of Vitamin C at the same concentration. Those results indicated that mannan from *Saccharomyces cerevisiae* has an intense free-radical scavenging ability, and was seriously influenced by their MW and concentration. According to Li et al. [27], the scavenging activity on hydroxyl radical was enhanced by the decreased MW of polysaccharides from *Enteromorpha prolifera*, which was similar to our results. In addition, there are some experiments concerning the mannans’ involvement with antitumor, antimetastatic, and hypolipidemic effects [28]. The antioxidant mechanisms may be due to the supply of hydrogen by polysaccharides, which could be combined with radicals to terminate the radical chain reaction.

### 3.5. Cytotoxicity of Mannans on Caco-2 Cells

**Figure 5:**
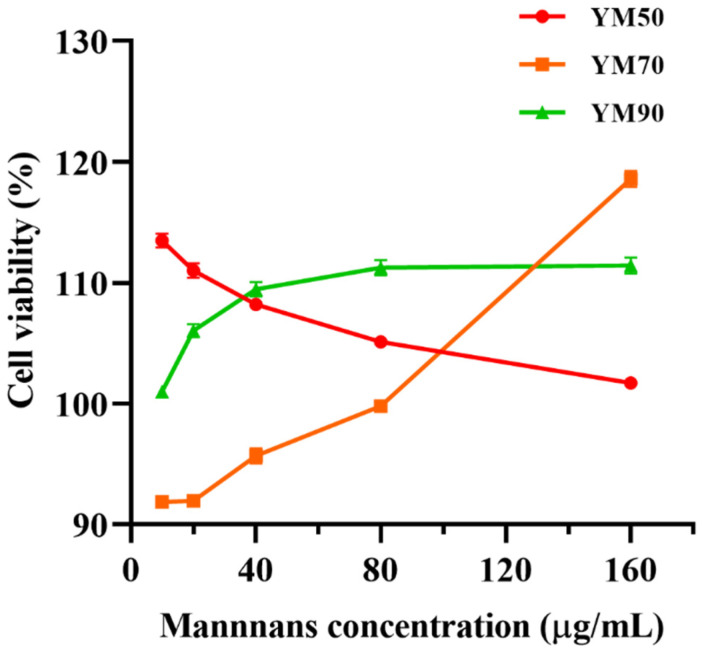
Cytotoxicity of mannans from on Caco-2 cells by MTT method. Data are mean ± SD (*n* = 6).

The cytotoxic effect of mannans of various molecular weights (MW) on Caco-2 cells was examined by an MTT assay. The results demonstrated that the toxicity of mannans was highly influenced by MW and polysaccharide concentration (Figure 5). All samples of YM90 and YM50 at concentrations as high as 160 μg/mL did not affect the relative viability of Caco-2 cells after 24 h exposure. In comparison, YM70 mannans led to a slightly limited toxicity on Caco-2 cells, with 95–99% relative viability at 10–80 μg/mL.

### 3.6. Hemolytic Activity of Mannans

**Figure 6:**
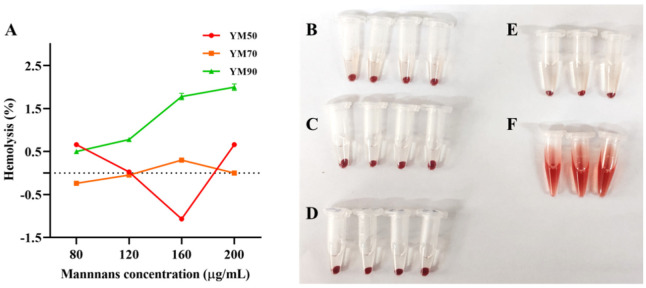
Hemolytic mannan assay. (**A**) Relative rate of hemolysis in mice erythrocytes upon incubation with mannans’ solution at incremental concentrations. The presence of hemoglobin in the supernatant was observed in (**B**) YM50 mannans, (**C**) YM70 mannans, (**D**) YM90 mannans, (**E**) negative control (PBS), and (**F**) positive control (water), respectively. Data are mean ± SD (*n* = 3).

The hemolytic activity of mannans with different MWs was tested on red blood cells (RBCs). The quantification of hemoglobin of mannans was carried out by recording the hemoglobin absorbance at 540 nm. The mannan samples were assayed at different concentrations (80–200 μg/mL), and the hemolysis assay showed that the hemolytic activity was molecular-weight- and concentration-dependent for mannans (Figure 6). For the tested YM50 and YM70 mannans, no hemolytic toxicity was observed below 200 μg/mL. The impact of polysaccharide geometry became pronounced as the molecular weight decreased. YM90 mannans caused an immediate onset of hemolysis, which soon reached a plateau of 2.0% hemolysis at ca.200 μg/mL, probably due to the porous architecture. This result suggested that the external surface area and the curvature of bioactive influence their hemolytic activity by affecting the magnitude of the binding energy of polysaccharides with RBCs, or the bending energy allowing the membrane to wrap around the polysaccharide [29]. A large external surface area and slight curvature rendered the hemolysis process thermodynamically favorable [30]. Hence, these results suggested that the mannans from *Saccharomyces cerevisiae* with different MWs (YM50-172.90 kDa, YM70-87.09 kDa, and YM90-54.05 kDa) were regarding as bio-safety.

## 4. Conclusions

In this research, purified mannans with different molecular weights (MW) were extracted from *Saccharomyces cerevisiae.* The high-MW YM50 and YM70 mannans showed a smooth-surface structure while the lowest MW YM90 mannans showed a rough surface with numerous cavities caused by SEM. The yeast mannans were shown to be in pyranose sugar forms by FTIR. The study may provide new insights into the digestion and distribution of mannans polysaccharides based on their microscopic morphologies and (bio)physicochemical properties. The in vitro antioxidant activity assay showed that mannans exhibited a valuable high scavenging activity for DPPH radicals and hydroxyl radicals in an MW- and concentration-dependent manner, indicating that the *Saccharomyces cerevisiae* polysaccharide may be a potential antioxidant and therapeutic agent. Slight cytotoxicity and hemolysis were found in all mannans, indicating they were bio-safe polysaccharides. These results provide a theoretical basis for further research on the absorption and application of the mannan polysaccharides with various MWs in vitro and in vivo. Further studies on the bioavailability and absorption mechanism in vivo of *Saccharomyces cerevisiae* polysaccharide mannans are ongoing in our lab.

## Figures and Tables

**Table 1:** Molecular weight distribution of mannans.

Mannan Samples	Molecular Weight (Da)	Filtration Volume (mL)	Partition Coefficient (K_av_)
native YM50	172,901	112.5	0.0917
native YM70	87,096	144	0.209
native YM90	54,050	166.5	0.294

## Data Availability

Not applicable.
